# High triglyceride–glucose index is associated with poor cardiovascular outcomes in Chinese acute coronary syndrome patients without diabetes mellitus who underwent emergency percutaneous coronary intervention with drug-eluting stents

**DOI:** 10.3389/fendo.2023.1101952

**Published:** 2023-02-15

**Authors:** Yong Zhang, Chao Chu, Zhong Zhong, Yong-bai Luo, Fei-fei Ning, Ning Guo

**Affiliations:** ^1^ Department of Cardiovascular Medicine, The First Affiliated Hospital of Xi’an Jiaotong University, Xi’an, Shaanxi, China; ^2^ Department of Cardiovascular Medicine, Weinan Central Hospital, Weinan, Shaanxi, China

**Keywords:** triglyceride-glucose index, insulin resistance, acute coronary syndrome, emergency percutaneous coronary intervention, major adverse cardiovascular and cerebrovascular event

## Abstract

**Background:**

Previous research has supported the association between the triglyceride–glucose index (TyG index) and the incidence and prognosis of cardiovascular disease. However, the association between the TyG index and the prognosis of patients with acute coronary syndrome (ACS) without diabetes mellitus (DM) who underwent emergency percutaneous coronary intervention (PCI) with drug-eluting stents (DESs) has not been thoroughly investigated, and these patients may easily be neglected. Therefore, this study aimed to investigate the association between the TyG index and major adverse cardiovascular and cerebrovascular events (MACCEs) in Chinese ACS patients without DM who underwent emergency PCI with DES.

**Methods:**

The total number of ACS patients without DM who underwent emergency PCI with DES for this study was 1650. Ln [fasting triglycerides (mg/dL) ×fasting plasma glucose (mg/dL)/2] is the formula used to calculate the TyG index. According to the TyG index, we classified the patients into two groups. The frequency of the following endpoint events was calculated and compared between the two groups: all-cause death, non-fatal myocardial infarction (MI), non-fatal ischemia stroke, ischemia-driven revascularization and cardiac rehospitalization.

**Results:**

After a median of 47 months of follow-up [47 (40, 54)], 437 (26.5%) endpoint events were recorded in total. The TyG index was further demonstrated to be independent of MACCE by multivariable Cox regression analysis (hazard ratio [HR], 1.493; 95% confidence interval [CI], 1.230–1.812; *p<*0.001). The TyG index≥7.08 group had a considerably greater incidence of MACCE (30.3% vs. 22.7% in the TyG index<7.08 group, *p*<0.001), cardiac death (4.0% vs. 2.3% in the TyG index<7.08 group, *p*=0.047), and ischemia-driven revascularization (5.7% vs. 3.6% in the TyG index<7.08 group, *p*=0.046) than the TyG index<7.08 group. Between the two groups, there was no discernible difference in all-cause death (5.6% vs. 3.8% in the TyG index<7.08 group, *p*=0.080), non-fatal MI (1.0% vs. 0.2% in the TyG index<7.08 group, *p*=0.057), non-fatal ischemic stroke (1.6% vs. 1.0% in the TyG index<7.08 group, *p*=0.272), and cardiac rehospitalization (16.5% vs. 14.1% in the TyG index<7.08 group, *p*=0.171).

**Conclusion:**

For ACS patients without DM who received emergency PCI with DES, the TyG index might be an independent predictor of MACCE.

## 1 Introduction

The most severe form of atherosclerotic cardiovascular disease (ASCVD), acute coronary syndrome (ACS), is responsible for the majority of cardiovascular disease (CVD)-related morbidity and mortality worldwide (1, 2). Since the significant development and implementation of superior evidence-based treatments, such as optimized medical therapy and revascularization, in recent years, the prognosis of ACS patients has noticeably improved. Nevertheless, the risk of recurrent poor cardiovascular events remains very high in patients with ACS (3, 4). As a result, clinical trials should identify the characteristics that put patients with ACS at a high risk of developing major adverse cardiovascular and cerebrovascular events (MACCEs), establish novel treatment targets, and develop tailored approaches that are line with risk levels.

Various variables, such as high glucose and lipid abnormalities, contribute to the development of CVD (5, 6). The primary pathophysiological underpinning for the beginning of ACS is thought to be the rupture and erosion of atherosclerotic plaque. Insulin resistance (IR) has been demonstrated to be a critical mediator in the development of metabolic disorders and atherosclerotic CVD (7, 8). Despite being widely acknowledged as the most accurate and trustworthy procedures to measure IR, the homeostasis model assessment of IR (HOMA-IR) and hyperinsulinemia-euglycemic clamp are complicated and costly to be applied in clinical practice (9). As a result, we require a straightforward, usable, and trustworthy marker to assess IR. The key elements of Mets are high levels of fasting blood glucose (FBG) and triglyceride (TG). The triglyceride–glucose (TyG) index, which is simply a combination of TG and FBG, has recently been revealed to have a substantial connection with the HOMA-IR and hyperinsulinemia-euglycemic clamp (10, 11). Furthermore, numerous studies have shown that the TyG index can indicate myocardial infarction (MI), stroke, or other adverse cardiovascular events (12–14). However, IR is easy to be ignored in non-diabetic patients. Studies have shown that IR is related to in-stent restenosis (ISR) in non-diabetic patients undergoing coronary stenting (15–17). The association between the TyG index and the prognosis of ACS patients without diabetes mellitus (DM) who underwent emergency percutaneous coronary intervention (PCI) with drug-eluting stents (DESs) has not been thoroughly investigated. Therefore, the purpose of the current study was to investigate the association between the TyG index and MACCEs in Chinese ACS patients without DM who underwent emergency PCI with DES.

## 2 Methods

### 2.1 Study population

**Figure 1:**
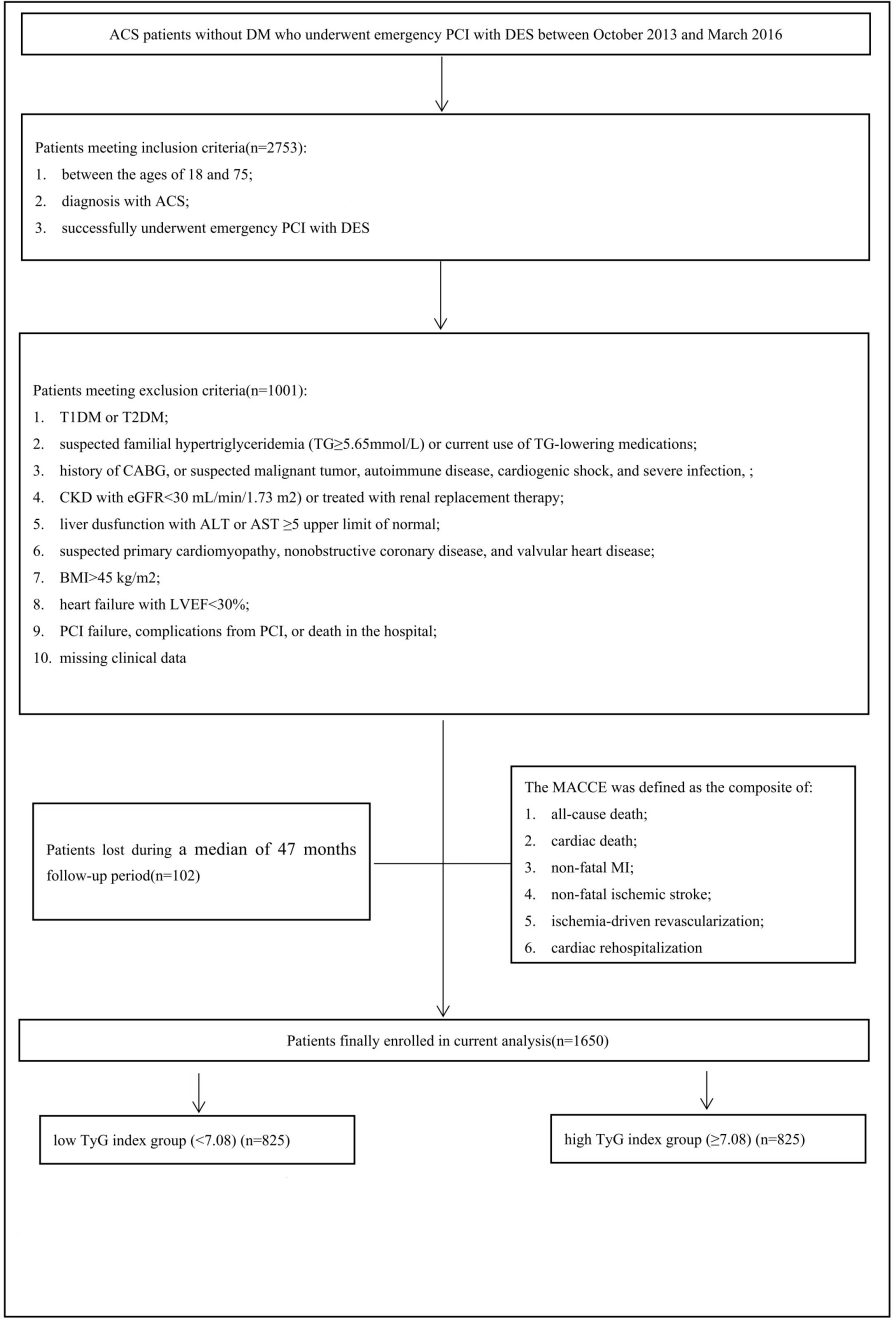
The flowchart of study subject enrollment. ACS, acute coronary syndrome PCI, percutaneous coronary intervention. DES, drug-eluting stent; TG, triglyceride; TIDM, type I diabetes mellitus; CABG, coronary artery bypass grafting; CKD, chronic kidney disease; eGFR, estimated glomerular filtration rate; ALT, alanine transaminase; AST, aspartate transaminase; BMI, body mass index, LVEF, left ventricular ejection fraction; MACCE, major adverse cardiovascular and cerebrovascular events, MI, myocardial infarction; TYG, triglyceride-glucose.

The current research was conducted between October 2013 and March 2016 on ACS patients without DM who received emergency PCI with DES at the First Affiliated Hospital of Xi’an Jiaotong University. It is an observational, retrospective cohort study with a single center. The following were the inclusion requirements: (1) between the ages of 18 and 75; (2) with an ACS diagnosis; and (3) successfully completed emergency PCI with DES. A total of 2753 consecutive ACS cases were examined. The following were the primary exclusion criteria for this study: (1) type 1 DM (T1DM) or type 2 DM (T2DM); (2) suspected familial hypertriglyceridemia (TG≥5.65 mmol/L) or current use of TG-lowering drugs; (3) history of coronary artery bypass grafting (CABG), or suspected malignant tumor, autoimmune disease, cardiogenic shock, and severe infection; (4) chronic kidney disease (CKD) with estimated glomerular filtration rate (eGFR) < 30mL/min/1.73 m^2^) or treated with renal replacement; (5) body mass index (BMI) > 45 kg/m^2^; (6) suspected primary cardiomyopathy, nonobstructive coronary disease, and valvular heart disease; (7) liver dysfunction with aspartate transaminase (AST) or alanine transaminase (ALT) ≥5 times upper limit of normal; (8) heart failure with left ventricular ejection fraction (LVEF) < 30%; (9) PCI failure, complications from PCI, or death in the hospital; and (10) absence of clinical data. The exclusion criteria resulted in the exclusion of 1101 patients. Over the duration of the follow-up period, 102 patients were lost. Finally, 1650 ACS patients were included in the study. According to the cohort’s median TyG index level (7.08), the patients were separated into two groups: low TyG index group (<7.08) and high TyG index group (≥7.08) (**Figure 1**).

### 2.2 Data collection and follow-up

Clinicians with the appropriate training gathered clinical data from the electronic medical records. These records contained information on people’s anthropometric characteristics, laboratory test results, and their medical and procedural histories. After fasting for the previous night, the venous blood samples were obtained in the morning and examined in the central laboratory on the same day according to established laboratory procedures. All patients were routinely observed by professional clinicians for MACCEs at 3, 6, and 12 months following hospitalization, as well as every 6 months for up to 66 months. Follow-up data were collected from hospital records or through telephone or in-person interviews with patients and their families.

MACCE, the observational endpoint of the current trial, was the composite of cardiac rehospitalization (admission because of angina or heart failure), ischemia-driven revascularization, non-fatal ischemic stroke, non-fatal myocardial infarction (MI), cardiac death, and all-cause death. Only the most serious event (all-cause death > non-fatal MI > non-fatal ischemic stroke > ischemia-driven revascularization > cardiac rehospitalization) was chosen for our analysis for patients with several poor outcomes almost occurring at the same time throughout the follow-up. Only the first instance of the same incident for patients with numerous occurrences was chosen for our analysis.

### 2.3 Definitions

The diagnostic criteria for ACS, which comprised ST-segment elevation myocardial infarction (STEMI) and non-ST-segment elevation acute coronary syndrome (NSTE-ACS: non-ST-segment elevation MI [NSTEMI] or unstable angina [UA]), were directed to appropriate guidelines (18, 19). Patients were considered to have hypertension if they had previously received a conclusive diagnosis or if their systolic blood pressure (SBP) ≥ 140 mmHg and/or diastolic blood pressure (DBP) ≥ 90 mmHg on more than two separate days during their initial hospitalization. According to the practical guidelines, patients with T1DM or T2DM were those who had a prior, conclusive diagnosis or who have just had their T1DM or T2DM confirmed (20). Patients were considered to have hypercholesterolemia if their fasting total cholesterol (TC) >6.22 mmol/L, their low-density lipoprotein cholesterol (LDL-C)>4.14 mmol/L, or they were on lipid-lowering medication. Patients with cerebral infarction or cerebral hemorrhage were considered to have a stroke. Patients with peripheral artery disease (PAD) were those who had artery disease that affected arteries other than the aorta and coronary arteries with stenosis ≥ 50% and related ischemic symptoms and/or signs. Patients were considered to have renal dysfunction if their eGFR was between 30 and 60mL/min/1.73m^2^. Emergency PCI was defined as PCI performed within 24 h of hospital admission for NSTE-ACS patients or within 12 h of symptom onset for STEMI patients.

The following equation was used to determine the TyG index: ln[fasting TG (mg/mL)×FBG (mg/mL)/2] (21). BMI was calculated as follows: weight (kg)/[height (m)]^2^. The formula for eGFR was 186×serum creatinine (mg/dL)^-1.154^×age^-0.203^ (×0.742 if female) (22).

A number of main coronary vessels, including left anterior descending artery, left circumflex artery, and right coronary artery, must have a stenosis ≥50% to be considered multivessel lesions. According to the prior medical history or coronary angiography, a chronic total occlusion (CTO) lesion was a complete obstruction lesion that lasted more than 3 months. A single stenotic lesion that was longer than 20 mm was considered a diffuse lesion. Stenosis of more than 50% appearing in the segment inside the stent, 5 mm proximal or distal to the stent, was described as ISR (23).

### 2.4 Statistical analysis

Continuous variables were presented as mean ± standard deviation (SD) or median. Comparisons between the two groups were analyzed by independent-sample *t*-test or Mann–Whitney *U*-test. Categorical variables were described as counts (percentages) and compared by Pearson chi-square test (Pearson ^2^ test) or Fisher’s exact test. The predictive value of the variables for MACCE was evaluated by univariate and multivariable Cox proportional hazards analysis. Several risk factors including important clinical and significant variables (p<0.2) in the univariate model were included in the multivariate. The cumulative incidence of MACCE was estimated by Kaplan–Meier survival curves. Further stratified analysis was conducted to determine the consistency of the prognostic impact of the TyG index for MACCE.

IBM SPSS Statistics (version 24.0) was used for data analysis. A two-tailed *p* value <0.05 was regarded as statistically significant.

## 3 Results

Final enrollment in the research included 1650 patients (60.50 ± 10.19 years; 22.5% female). A total of 77 (4.7%) all-cause deaths including 52 (3.1%) cardiac deaths, 10 (0.6%) non-fatal MIs, 21 (1.3%) non-fatal ischemic strokes, 77 (4.7%) ischemia-driven revascularizations, and 252 (15.3%) cardiac rehospitalizations occurred throughout the duration of the 66-month follow-up. Finally, 437 (26.5%) MACCEs in total were included in the analysis.

### 3.1 Baseline characteristics of the low and high TyG index groups

According to the median TyG index level (7.08) of the cohort, the patients were separated into two groups: low TyG index group (<7.08) and high TyG index group (≥7.08). **Table 1** shows the baseline characteristics of the two groups. Patients in the high TyG index group had a faster heart rate (74.53 ± 12.11 vs. 73.29 ± 11.96, *p*=0.036), higher percentage of males (80.1% vs. 74.9%, *p*=0.011), higher previous history of hyperlipidemia (15.3% vs. 6.5%, *p*<0.001), and lower percentage of smoking (52.6% vs. 59.9%, *p*=0.003) than those in the low TyG index group. In terms of laboratory findings, patients in the high TyG index group had raised levels of white blood cell (WBC; 7.46 ± 2.54 vs. 7.14 ± 2.58, *p*=0.013), hemoglobin (Hb; 139.51 ± 18.30 vs. 136.79 ± 18.41, *p*=0.003), blood urea nitrogen (BUN; 5.46 ± 1.70 vs. 5.13 ± 1.93, *p*<0.001), FBG (6.64 ± 2.07 vs. 5.19 ± 1.24, *p*<0.001), glycosylated hemoglobin A1c (HbA1c; 5.74 ± 0.70 vs. 5.49 ± 0.54, *p*<0.001), TC (4.01 ± 1.16 vs. 3.41 ± 1.09, *p*<0.001), TG (1.98 ± 1.05 vs. 1.07 ± 0.33, *p<*0.001), and LDL-C (1.96 ± 0.95 vs. 1.67 ± 0.70, *p*<0.001) but decreased level of high-density lipoprotein cholesterol (HDL-C; 0.95 ± 0.22 vs. 1.00 ± 0.23, *p*<0.001). No differences were found between the two groups in terms of coronary intervention information and medication at discharge.

**Table 1:** Baseline clinical characteristics of patients in the low and high TyG index groups.

Characteristics	Total population	Low TyG index group	High TyG index group	*P* value
	(n=1650)	(n=825)	(n=825)	
Age, years	60.50 ± 10.19	61.14 ± 10.34	59.85 ± 10.01	0.010
Gender, male, n (%)	1279 (77.5%)	618 (74.9%)	661(80.1%)	0.011
BMI, kg/m2	23.26 ± 2.46	23.36 ± 2.48	23.17 ± 2.43	0.118
SBP, mmHg	126.93 ± 20.74	126.46 ± 20.96	127.39 ± 20.51	0.363
DBP, mmHg	77.67 ± 11.97	77.28 ± 11.87	78.06 ± 12.07	0.188
Heart rate, bpm	73.91 ± 12.05	73.29 ± 11.96	74.53 ± 12.11	0.036
Smoking history, n (%)	928(56.2%)	494(59.9%)	434(52.6%)	0.003
Drinking history, n (%)	459(27.8%)	239(29.0%)	220(26.7%)	0.297
Family history of CAD, n (%)	145(8.8%)	70(8.5%)	75(9.1%)	0.664
Initial diagnosis, n (%)				0.134
UA	976(59.2%)	492(59.6%)	484(58.7%)	
NSTEMI	118(7.2%)	68(8.2%)	50(6.1%)	
STEMI	556(33.7%)	265(32.1%)	291(35.3%)	
Medical history, n (%)				
Hypertension	886(53.7%)	434(52.6%)	452(54.8%)	0.374
Hyperlipidemia	180(10.9%)	54(6.5%)	126(15.3%)	< 0.001
Renal dysfunction	14(0.8%)	7(0.8%)	7(0.8%)	> 0.999
Previous MI	148(9.0%)	77(9.3%)	71(8.6%)	0.605
Previous PCI	158(9.6%)	86(10.4%)	72(8.7%)	0.241
Previous stroke	321(19.5%)	157(19.0%)	164(19.9%)	0.663
Previous PAD	260(15.8%)	119(14.4%)	141(17.1%)	0.137
Laboratory results				
WBC (×109/L)	7.30 ± 2.57	7.14 ± 2.58	7.46 ± 2.54	0.013
PLT (×109/L)	158.49 ± 55.42	156.37 ± 53.36	160.61 ± 57.35	0.120
Hb (g/L)	138.15 ± 18.40	136.79 ± 18.41	139.51 ± 18.30	0.003
BUN (mmol/L)	5.29 ± 1.83	5.13 ± 1.93	5.46 ± 1.70	< 0.001
Cr (umol/L)	67.93 ± 17.72	67.81 ± 18.22	68.05 ± 17.21	0.779
eGFR (mL/min/1.73m2)	97.48 ± 27.25	96.98 ± 26.14	97.98 ± 28.34	0.455
FBG (mmol/L)	5.92 ± 1.85	5.19 ± 1.24	6.64 ± 2.07	< 0.001
HbA1C (%)	5.61 ± 0.64	5.49 ± 0.54	5.74 ± 0.70	< 0.001
HDL-C (mmol/L)	0.98 ± 0.23	1.00 ± 0.23	0.95 ± 0.22	< 0.001
TC (mmol/L)	3.71 ± 1.17	3.41 ± 1.09	4.01 ± 1.16	< 0.001
TG (mmol/L)	1.52 ± 0.90	1.07 ± 0.33	1.98 ± 1.05	< 0.001
LDL-C (mmol/L)	1.82 ± 0.85	1.67 ± 0.70	1.96 ± 0.95	< 0.001
NT-proBNP (pg/mL)	690.55 ± 1233.19	729.52 ± 1239.91	651.57 ± 1225.95	0.199
LVEF (%)	60.07 ± 11.28	59.99 ± 11.21	60.16 ± 11.36	0.760
Angiographic data				
LM disease, n (%)	174(10.5%)	88(10.7%)	86(10.4%)	0.873
CTO, n (%)	475(28.8%)	243(29.5%)	232(28.8%)	0.550
Number-vessel disease. n (%)				0.574
Single-vessel disease	445(27.0%)	231(28.0%)	214(25.9%)	
Two-vessel disease	497(30.1%)	249(30.2%)	248(30.1%)	
Three-vessel disease	708(42.9%)	345(41.8%)	363(44.0%)	
Diffuse lesion, n (%)	985(59.7%)	506(61.3%)	479(58.1%)	0.175
In-stent restenosis, n (%)	48(2.9%)	27(3.3%)	21(2.5%)	0.379
Calcification lesion, n (%)	38(2.3%)	18(2.2%)	20(2.4%)	0.743
Number of stents	1.71 ± 1.15	1.68 ± 1.11	1.74 ± 1.20	0.276
Medication at discharge, n (%)				
ACEI/ARB	1345(81.5%)	664(80.5%)	681(82.5%)	0.281
β-blocker	1338(81.1%)	663(80.4%)	675(81.8%)	0.451
Statins	1643(99.6%)	824(99.9%)	819(99.3%)	0.124
P2Y12 inhibitor				0.934
Clopidogrel	1491(90.4%)	746(90.4%)	745(90.3%)	
Ticagrelor	159(9.6%)	79(9.6%)	80(9.7%)	
Aspirin	1650(100.0%)	825(100%)	825(100%)	–
DAPT	1650(100.0%)	825(100%)	825(100%)	–

TyG, triglyceride-glucose; BMI, body mass index; SBP, systolic blood pressure; DBP, diastolic blood pressure; CAD, coronary artery disease; UA, unstable angina; NSTEMI, non ST-segment elevation myocardial infarction; STEMI, ST-segment elevation myocardial infarction; MI, myocardial infarction; PCI, percutaneous coronary intervention; PAD, peripheral artery disease; WBC, white blood cell; PLT, platelet; Hb, hemoglobin; BUN, blood urea nitrogen; Cr, creatinine; eGFR, estimated glomerular filtration rate; FBG, fasting blood glucose; HbA1C, glycosylated hemoglobin A1c; HDL-C, high-density lipoprotein cholesterol; TC, total cholesterol; TG, triglyceride; LDL-C, low-density lipoprotein cholesterol; LVEF, left ventricular ejection fraction; LM, left main; CTO, chronic total occlusion; ACEI, angiotensin converting enzyme inhibitor; ARB, angiotensin receptor blocker; DAPT, dual antiplatelet therapy.

### 3.2 Predictive implications of the TyG index for MACCE

**Figure 2:**
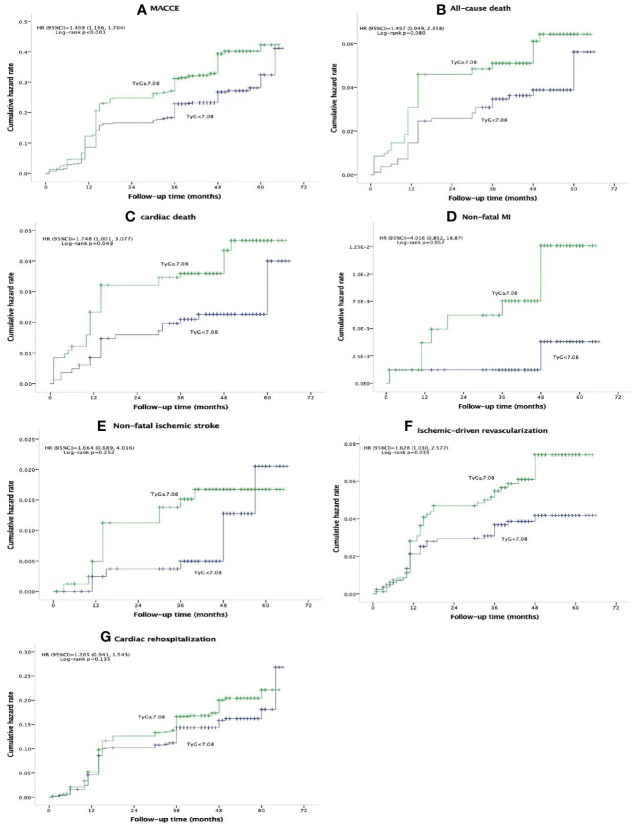
Kaplan-Meier curves for composite MACCE **(A)**, all-cause death **(B)**, cardiac death **(C)**, non-fatal MI **(D)**, non-fatal ischemic stroke **(E)**, schemic-driven revascularization **(F)**, and cardiac rehospitalization **(G)** of the TyG<7.08 group (blue line) versus the TyG<7.08 group (green line). TYG, triglyceride-glucose index; MACCE, major adverse cardiovascular and cerebrovascular event; MI, myocardial infarction; HR, hazard ratio; Cl, confidence interval.

There were 437 (26.5%) MACCEs over the course of a median of 47 months follow-up [47 (40, 54)], including 77 (4.7%) all-cause deaths, 52 (3.2%) cardiac deaths, 10 (0.6%) non-fatal MIs, 21 (1.3%) non-fatal ischemic strokes, 77 (4.7%) ischemia-driven revascularizations, and 252 (15.3%) cardiac rehospitalizations. Patients in the high TyG index group had significantly greater incidences of MACCE (30.3% vs. 22.7%, *p*<0.001), cardiac death (4.0% vs. 2.3%, *p*=0.047), and ischemia-driven revascularization (5.7% vs. 3.6%, *p*=0.046) compared with those in the low TyG index group. However, there was no significant difference in the prevalence of all-cause death (5.6% vs. 3.8%, *p*=0.080), non-fatal MI (1.0% vs. 0.2%, *p*=0.057), non-fatal ischemic stroke (1.6% vs. 1.0%, *p*=0.272), and cardiac rehospitalization (16.5% vs. 14.1%, *p*=0.171) between the two groups (**Table 2**). **Figure 2** displays the Kaplan–Meier curves for the incidence of MACCE and each of its components for the low and high TyG groups. The difference between the low and high TyG index groups was clearly visible in the Kaplan–Meier curves for MACCE (**Figure 2A**; hazard ratio [HR] 1.408, 95% confidence interval [CI] 1.166–1.704, log-rank *p*<0.001). The difference was mostly due to a rise in cardiac death (**Figure 2C**; HR 1.748, 95% CI 1.001–3.077, log-rank *p*=0.049), and ischemia-driven revascularization (**Figure 2F**; HR 1.628, 95% CI 1.030–2.577, log-rank *p*=0.035). A statistically significant difference between the low and high TyG index groups could not be found in the Kaplan–Meier curves for all-cause death (**Figure 2B**; HR 1.497, 95% CI 0.949–2.358, log-rank *p*=0.080), non-fatal MI (**Figure 2D**; HR 4.016, 95% CI 0.852–18.87, log-rank *p*=0.057), non-fatal ischemic stroke (**Figure 2E**; HR 1.664, 95% CI 0.689–4.016, log-rank *p*=0.252), and cardiac rehospitalization (**Figure 2G**; HR 1.205, 95% CI 0.941–1.543, log-rank *p*=0.135).

**Table 2:** Incidence of MACCE in low and high TyG index group.

Endpoint event	Total population	Low TyG index group	High TyG index group	*P* value
	(n=1650)	(n=825)	(n=825)	
MACCE, n (%)	437(26.5%)	187(22.7%)	250(30.3%)	< 0.001
All-cause death, n (%)	77(4.7%)	31(3.8%)	46(5.6%)	0.080
Cardiac death, n (%)	52(3.2%)	19(2.3%)	33(4.0%)	0.047
Non-fatal MI, n (%)	10(0.6%)	2(0.2%)	8(1.0%)	0.057
Non-fatal ischemic stroke, n (%)	21(1.3%)	8(1.0%)	13(1.6%)	0.272
Ischemia-driven revacularization, n (%)	77(4.7%)	30(3.6%)	47(5.7%)	0.046
Cardiac rehospitalization, n (%)	252(15.3%)	116(14.1%)	136(16.5%)	0.171

MACCE, major adverse cardiovascular and cerebrovascular events; TyG, triglyceride-glucose; MI, myocardial infarction.

### 3.3 Cox proportional hazard analysis to evaluate the prognostic implication of MACCEs

The relationship between the TyG index and MACCEs was analyzed using the Cox proportional hazards model. Univariate analysis revealed that the TyG index was substantially related to MACCEs (HR 1.408, 95% CI 1.166–1.704, *p*<0.001). Gender, STEMI, drinking history, number of stents implanted, left main (LM) disease, two-vessel and three-vessel disease, Hb, FBG, TG, and β-blocker medication at discharge were the additional significant risk variables. Several risk factors including important clinical and significant variables (*p*<0.05) in the univariate model were included in the multivariate.The TyG index continued to be an independent predictor of MACCEs (HR 1.493, 95% CI 1.230–1.812, *p<*0.001). STEMI, hyperlipidemia, Hb, and β-blocker medication at discharge were the additional independent predictors of MACCEs in the multivariate analysis (**Table 3**).

**Table 3:** Predictive value of TyG index for MACCE in univariate and multivariate analysis.

Characteristics	Univariate analysis		Multivariate analysis	
	HR (95%CI)	*P* value	HR(95%CI)	*P* value
Age	1.006(0.997, 1.016)	0.195		
Gender	1.240(1.001, 1.537)	0.049	1.068(0.824, 1.385)	0.616
Previous MI	1.224(0.899, 1.666)	0.199		
Previous PCI	1.289(0.960, 1.731)	0.091		
Initial diagnosis		0.010		0.007
UA	reference		reference	
NSTEMI	1.069(0.705, 1.621)	0.753	1.094(0.720, 1.662)	0.674
STEMI	1.372(1.111, 1.695)	0.003	1.399(1.131, 1.731)	0.002
Hyperlipidemia	1.377(0.984, 1.931)	0.062	1.481(1.052, 2.083)	0.025
Smoking history	1.202(0.996, 1.451)	0.054		
Drinking history	0.769(0.616, 0.960)	0.020	0.843(0.667, 1.065)	0.153
HR	1.006(0.998, 1.013)	0.140		
Number-vessel disease		0.045		0.141
Single-vessel disease	reference		reference	
Two-vessel disease	1.266(1.012, 1.585)	0.042	1.200(0.944, 1.527)	0.135
Three-vessel disease	1.274(1.009, 1.608)	0.039	1.230(0.978, 1.548)	0.076
LM disease	1.361(1.033, 1.795)	0.029	1.290(0.971, 1.713)	0.079
Number of stents	1.096(1.014, 1.185)	0.021	1.060(0.978, 1.150)	0.158
WBC	1.026(0.998, 1.027)	0.174		
Hb	0.993(0.989, 0.998)	0.010	0.994(0.988, 0.999)	0.036
HDL-C	0.702(0.467, 1.056)	0.089		
TyG index	1.408(1.166, 1.704)	< 0.001	1.493(1.230, 1.812)	<0.001
β-blocker medication	0.761(0.588, 0.986)	0.039	0.759(0.585, 0.984)	0.037

TyG, triglyceride-glucose; MACCE, major adverse cardiovascular and cerebrovascular events; HR, hazard ratio; CI, confidence interval; MI, myocardial infarction; PCI, percutaneous coronary intervention; UA, unstable angina; NSTEMI, non ST-segment elevation myocardial infarction; STEMI, ST-segment elevation myocardial infarction; HR, heart rate; LM, left main; WBC, white blood cell; Hb, hemoglobin; FBG, fasting blood glucose; HDL-C, high-density lipoprotein cholesterol; TG, triglyceride.

### 3.4 Sensitivity analysis

**Figure 3:**
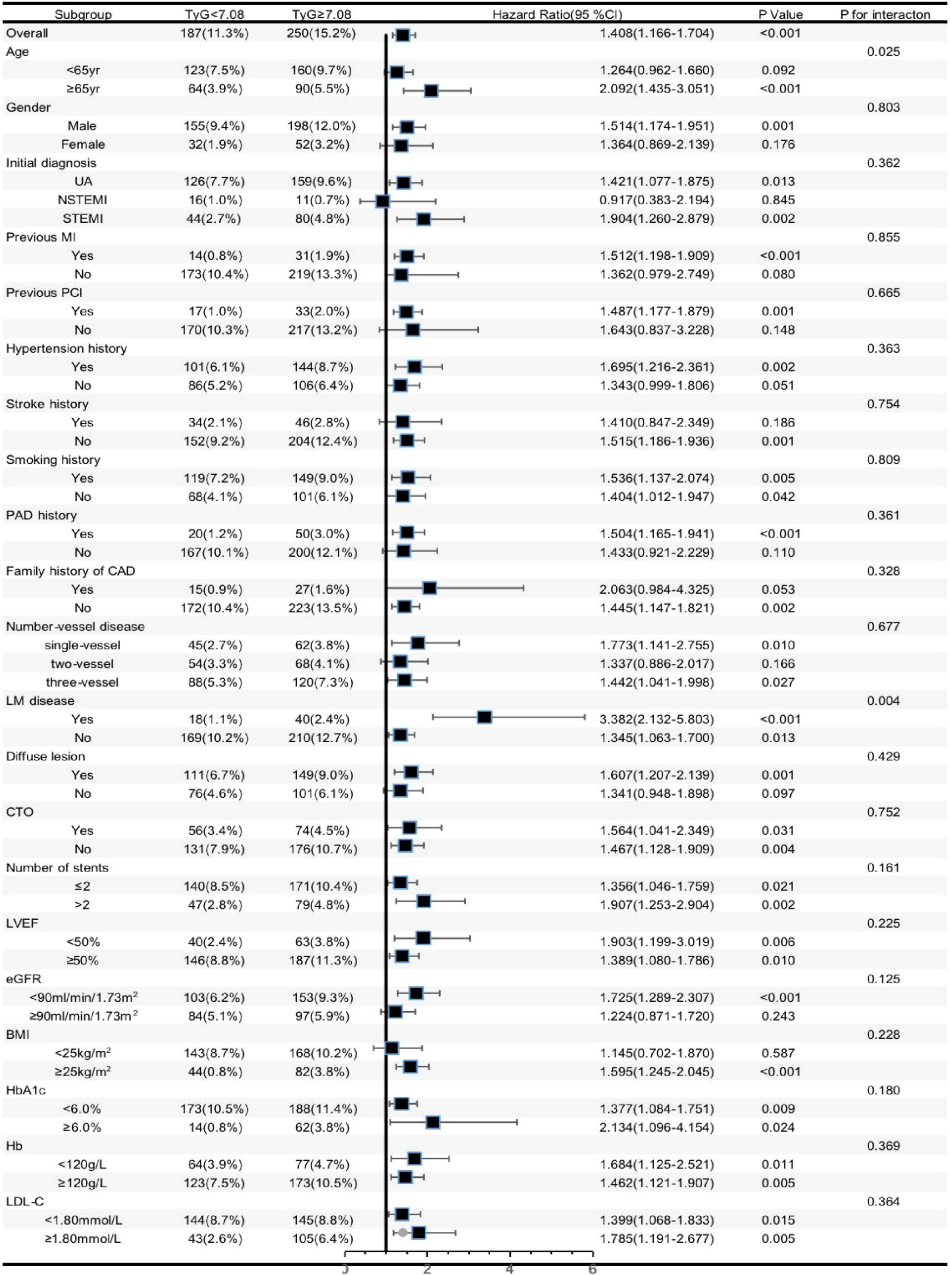
Forest plot investigating the association between the TyG index and MACCE in different subgroups. TYG, triglyceride-glucose index; UA, unstable angina, NSTEMI, non ST-segment elevation myocardial infarction; STEMI, ST-segment elevation myocardial infarction; MI, myocardial infarction; PCI, percutaneous coronary intervention; DM, diabetes mellitus; CAD, coronary artery disease; LM, left main; CTO, chronic total occlusion; Hb, hemoglobin;LVEF, left ventricular ejection fraction; eGFR, estimated glomerular filtration rate; BMI, body mass index; HbA1c, glycosylated hemoglobin A1c; HDL-C, high-density lipoprotein cholesterol, LDL-C, low-density lipoprotein cholesterol; C, confidence interval.

We conducted additional analysis among several subgroups to assess the independent relationship of the TyG index with MACCE. As shown in **Figure 3**, the independent predictive effect of the TyG index on MACCE was primarily represented in the subgroups of male, age≥65 years, UAP and STEMI, with previous MI history, with previous PCI history, with hypertension history, without stroke history, with and without smoking history, with previous PAD history, without family history of CAD, single-vessel disease and three-vessel disease, with and without LM disease, with diffuse lesion group, with and without CTO, ≤2 and >2 stents implanted, LVEF≥50% and <50%, eGFR < 90mL/min/1.73 m^2^, BMI ≥25 kg/m^2^, HbA1c <6.0% and ≥6.0%, Hb≥120 g/L, and LDL-C <1.80 mmol/L and≥1.80 mmol/L. Interestingly, patients≥65 years of age (HR [95%CI] age≥65 years 2.092 (1.435–3.051) vs. age<65 years 1.264 (0.962–1.660), *p* for interaction =0.025) and those with LM disease (HR [95%CI] with LM disease 3.382 (2.132–5.803) vs. without LM disease 1.345 (1.063–1.700), *p* for interaction =0.004] appeared to have higher predictive values for the TyG index.

## 4 Discussion

The high TyG index group had a considerably higher incidence of MACCE than the low TyG index group in this cohort of Chinese ACS patients without DM who underwent emergency PCI with DES. The TyG index is an independent predictor of MACCEs. This work is the first study that investigated the association of between the TyG index and the prognosis of the Chinese ACS patients without DM who underwent emergency PCI with DES.

The knowledge of atherogenesis has advanced significantly. The advancement of atherosclerosis and cardiovascular events is related to LDL-C levels. Using statins (24), ezetimibe (25), and proprotein convertase subtilisin/kexin type9 (PCSK9) antibodies (26) to lower LDL-C levels can reduce risk by about 30%–50%; however, a significant amount of residual risk remains (24). Cardiologists that specialize in coronary heart disease have recently focused on inflammation inhibition. CANTOS (Canakinumab Anti-inflammatory Thrombosis Outcomes Study) demonstrated for the first time that inhibiting the interleukin (IL)-1b pathway can lower the risk of cardiovascular events in patients with coronary artery disease (CAD) by about 17% (27). As one of the major risk factors for CVD, DM may be treated with hypoglycemic medications such as sodium-dependent glucose transporter 2 inhibitor (SGLT2i) and glucagon-like peptide-1 (GLP-1) receptor agonist to lower cardiovascular events (28). Therefore, the additional risk factors for MACCE in patients with ACS must be recognized. In our study, patients who belonged to the MACCE group had a perfect correlation with older age; initial diagnosis of STEMI; lower Hb; higher FBG, TG, and TyG index; more LM disease; more stents implanted, and low proportion of β-blocker medication at discharge (**Supplemental Table 1**). Cox multivariate analysis revealed that the TyG index, STEMI, hyperlipidemia, Hb, and β-blocker medication at discharge were all substantially correlated with MACCE.

IR has a significant connection to dyslipidemia and inflammation (29), but it has not been given much attention. A significant portion of the residual risk following lipid-lowering treatment may be due to IR (30–32). A composite indicator composed of TG and FBG is named the TyG index. In clinical practice, the TyG index is frequently used as a straightforward index to assess the degree of IR. Furthermore, the TyG index has a high level of sensitivity and specificity as a marker for IR (21, 33). According to our study, patients in the high TyG index group had higher levels of LDL-C, TC, TG and WBC; lower levels of HDL-C; and a higher percentage of prior history of hyperlipidemia than those in the low TyG index group.

In terms of the underlying mechanisms, previous research has suggested that IR may have a role in the emergence of adverse cardiovascular events (34, 35). First, catecholamine secretion, sympathetic activity, and myocardial oxygen consumption may all rise as a result of IR. Second, the renin-angiotensin-aldosterone system (RAAS) to may be activated by IR, which results in cardiac insufficiency. Third, IR may enhance the synthesis of coagulation and inflammatory factors, causing an imbalance in coagulation and abnormalities of the fibrinolytic system, which can result in thrombosis. Fourth, cardiomyopathy and heart failure may result from changes in heart metabolism and impaired systolic function caused by IR. Fifth, IR may increase the risk of major adverse cardiovascular events (MACEs) by promoting atherosclerosis and causing plaque instability (34, 36).

Extensive research has been made on the TyG index. A strong correlation between high TyG index and cardiovascular events has also been demonstrated by a number of sizable prospective trials. High TyG index was found to be significantly related to an elevated risk of CVD in a study that included 5,014 people who appeared to be in good health (37). The TyG index and carotid intima media thickness were found to be related in a study of 473 postmenopausal non-diabetic women (38). Additionally, the TyG index can predict the ischemic stroke risk in the general population (39). Thomas B et al. reported that the TyG index may be beneficial in determining people who are at high risk for CVD (40). The findings of this research, which investigated thousands of people for sufficient sample size and average duration of 10 years, revealed that higher TyG index levels were linked to an increased likelihood of CVD events and were independent prognostic factors, regardless of fundamental information including weight, gender, smoking, accompanying diseases (such as hypertension and T2DM), and general cardiovascular risk factors such as HDL-C and LDL-C. The findings demonstrated the value of the TyG index as a CAD predictor. Additional research has revealed that individuals with metabolic syndrome and CAD have a poor prognosis, with an increased risk of MACE and much more severe CAD (41). The TyG index has also been linked to short-term MACE and mortality risk in individuals who have undergone PCI (42). According to Laxy M.’s research, STEMI individuals with a high TyG index are more likely to experience a higher incidence of cardiac death, malignant arrhythmias, unstable angina, and heart failure rehospitalization during 2 years following PCI (43). The TyG index is also associated with cardiovascular events in non-diabetic patients. According to a previous study by Quiroga B, et al., the TyG index is associated with the incidence of cardiovascular events in non-diabetic patients with non-dialysis-dependent CKD (44). A study of elder non-diabetic patients with STEMI showed that the TyG index can predict in-hospital and long-term mortality (45). Therefore, the TyG index is a predictor of adverse events in healthy people, patients with CVD or undergoing PCI, and patients with or without DM. To assess the effectiveness of the TyG index for predicting adverse cardiovascular and cerebrovascular events in Chinese ACS patients without DM who underwent emergency PCI with DES, we performed an analysis based on a cohort of 1650 ACS patients without DM. We discovered that the prevalence of MACCE in the high TyG index group was considerably greater compared with that in the low TyG index group. Thus, the TyG index is an independent prognostic factor of MACCEs, and it plays a key role in subgroup analysis.

This study has a number of limitations that must be acknowledged. First, this work is retrospective, observational, and single center in design. As a result, this study was unable to establish a causal relationship between the TyG index and MACCE. Second, both the sample size and follow-up period were insufficient. Third, the fact that only patients with ACS who received emergency PCI with DES were included in the current study may have led to selection bias and restricted the generalizability of our findings to patients with ACS who underwent elective PCI or were treated with drug-coated balloon and patients with chronic coronary syndrome. Fourth, despite adjusting for the usage of lipid-lowering treatment, the results may be skewed as we did not consider the type, intensity, and variations in those medications. Fifth, minimal information was available regarding the change in the TyG index level during the follow-up period. Moreover, the laboratory data and TyG index were only examined once, both of which could have resulted in bias because of measurement error.

## 5 Conclusion

In Chinese ACS patients without DM who underwent emergency PCI with DES, increasing IR extent as shown by the TyG index is a prominent risk factor of MACCE. The identification of the TyG index may help with early classification and intervention to prevent MACCE. To support our results, additional multi-center, large-scale, and prospective cohort trials with a sizable sample and prolonged follow-up period are required.

## Data availability statement

The original contributions presented in the study are included in the article/**Supplementary Material**. Further inquiries can be directed to the corresponding author.

## Ethics statement

The studies involving human participants were reviewed and approved by the Academic Committee of the First Affiliated Hospital of Xi’an Jiaotong University. The patients/participants provided their written informed consent to participate in this study.

## Author contributions

YZ and NG were responsible for designing the study and writing of the manuscript. YZ, ZZ, and Y-BL performed the follow-up of this cohort and collected the data. CC and F-FN analyzed the data and revised the manuscript. YZ, ZZ, and F-FN were responsible for research organization and coordination. All authors contributed to the article and approved the submitted version.

## References

[1] Eisen A Giugliano RP Braunwald E. Updates on acute coronary syndrome: A review. JAMA Cardiol (2016) 1:718–30. doi: 10.1001/jamacardio.2016.2049 27438381

[2] Benjamin EJ Virani SS Callaway CW Chamberlain AM Chang AR Cheng S. Heart disease and stroke statistics-2018 update: A report from the American heart association. Circulation. (2018) 137:e67–e492. doi: 10.1161/cir.0000000000000558 29386200

[3] Jernberg T Hasvold P Henriksson M Hjelm H Thuresson M Janzon M. Cardiovascular risk in post-myocardial infarction patients: nationwide realworld data demonstrate the importance of a long-term perspective. Eur Heart J (2015) 36:1163–70. doi: 10.1093/eurheartj/ehu505 25586123

[4] Fox KA Carruthers KF Dunbar DR Graham C Manning JR De Raedt H. Underestimated and under-recognized: The late consequences of acute coronary syndrome (GRACE UK-Belgian study). Eur Heart J (2010) 31:2755–64. doi: 10.1093/eurheartj/ehq326 20805110

[5] Bays HE. Adiposopathy, diabetes mellitus, and primary prevention of atherosclerotic coronary artery disease: Treating "sick fat" through improving fat function with antidiabetes therapies. Am J Cardiol (2012) 110:4b–12b. doi: 10.1016/j.amjcard.2012.08.029 23062567

[6] Li XL Guo YL Zhu CG Xu RX Qing P Wu NQ. Relationship of high-density lipoprotein cholesterol with periprocedural myocardial injury following elective percutaneous coronary intervention in patients with low-density lipoprotein cholesterol below 70 mg/dL. J Am Heart Assoc (2015) 4:e001412. doi: 10.1161/jaha.114.001412 25572484 PMC4330066

[7] Ormazabal V Nair S Elfeky O Aguayo C Salomon C Zuñiga FA. Association between insulin resistance and the development of cardiovascular disease. Cardiovasc Diabetol (2018) 17:122. doi: 10.1186/s12933-018-0762-4 30170598 PMC6119242

[8] Di Pino A DeFronzo RA. Insulin resistance and atherosclerosis: Implications for insulin-sensitizing agents. Endocr Rev (2019) 40:1447–67. doi: 10.1210/er.2018-00141 PMC7445419 31050706

[9] Muniyappa R Lee S Chen H Quon MJ. Current approaches for assessing insulin sensitivity and resistance *in vivo*: Advantages, limitations, and appropriate usage. Am J Physiol Endocrinol Metab (2008) 294:E15–26. doi: 10.1152/ajpendo.00645.2007 17957034

[10] Du T Yuan G Zhang M Zhou X Sun X Yu X. Clinical usefulness of lipid ratios, visceral adiposity indicators, and the triglycerides and glucose index as risk markers of insulin resistance. Cardiovasc Diabetol (2014) 13:146. doi: 10.1186/s12933-014-0146-3 25326814 PMC4209231

[11] Navarro-González D Sánchez-Íñigo L Pastrana-Delgado J Fernández-Montero A Martinez JA. Triglyceride-glucose index (TyG index) in comparison with fasting plasma glucose improved diabetes prediction in patients with normal fasting glucose: The vascular-metabolic CUN cohort. Prev Med (2016) 86:99–105. doi: 10.1016/j.ypmed.2016.01.022 26854766

[12] Wang A Wang G Liu Q Zuo Y Chen S Tao B. Triglyceride-glucose index and the risk of stroke and its subtypes in the general population: An 11-year follow-up. Cardiovasc Diabetol (2021) 20:46. doi: 10.1186/s12933-021-01238-1 33602208 PMC7893902

[13] Tian X Zuo Y Chen S Liu Q Tao B Wu S. Triglyceride-glucose index is associated with the risk of myocardial infarction: An 11-year prospective study in the kailuan cohort. Cardiovasc Diabetol (2021) 20:19. doi: 10.1186/s12933-020-01210-5 33435964 PMC7802156

[14] Luo JW Duan WH Yu YQ Song L Shi DZ. Prognostic significance of triglyceride-glucose index for adverse cardiovascular events in patients with coronary artery disease: A systematic review and meta-analysis. Front Cardiovasc Med (2021) 8:774781. doi: 10.3389/fcvm.2021.774781 34926622 PMC8674619

[15] Radke PW Voswinkel M Reith M Kaiser A Haager PK Hanrath P. Relation of fasting insulin plasma levels to restenosis after elective coronary stent implantation in patients without diabetes mellitus. Am J Cardiol (2004) 93:639–41. doi: 10.1016/j.amjcard.2003.11.039 14996599

[16] Piatti P Di Mario C Monti LD Fragasso G Sgura F Caumo A. Association of insulin resistance, hyperleptinemia, and impaired nitric oxide release with in-stent restenosis in patients undergoing coronary stenting. Circulation. (2003) 108:2074–81. doi: 10.1161/01.Cir.0000095272.67948.17 14530196

[17] Hwang IK Kim YK Rha SW Ra JE Seo BS Lee JK. Impact of insulin resistance on 1-year clinical outcomes in non-diabetic patients undergoing percutaneous coronary intervention with drug-eluting stents. J Cardiol (2013) 61:113–6. doi: 10.1016/j.jjcc.2012.08.022 23159207

[18] Ibanez B James S Agewall S Antunes MJ Bucciarelli-Ducci C Bueno H. 2017 ESC Guidelines for the management of acute myocardial infarction in patients presenting with ST-segment elevation: The task force for the management of acute myocardial infarction in patients presenting with ST-segment elevation of the European society of cardiology (ESC). Eur Heart J (2018) 39:119–77. doi: 10.1093/eurheartj/ehx393 28886621

[19] Roffi M Patrono C Collet JP Mueller C Valgimigli M Andreotti F. 2015 ESC Guidelines for the management of acute coronary syndromes in patients presenting without persistent ST-segment elevation: Task force for the management of acute coronary syndromes in patients presenting without persistent ST-segment elevation of the European society of cardiology (ESC). Eur Heart J (2016) 37:267–315. doi: 10.1093/eurheartj/ehv320 26320110

[20] Association Diabetes Association. Diagnosis and classification of diabetes mellitus. Diabetes Care (2011) 34 Suppl 1:S62–9. doi: 10.2337/dc11-S062 PMC3006051 21193628

[21] Guerrero-Romero F Simental-Mendía LE González-Ortiz M Martínez-Abundis E Ramos-Zavala MG Hernández-González SO. The product of triglycerides and glucose, a simple measure of insulin sensitivity. comparison with the euglycemic-hyperinsulinemic clamp. J Clin Endocrinol Metab (2010) 95:3347–51. doi: 10.1210/jc.2010-0288 20484475

[22] Levey AS Coresh J Greene T Stevens LA Zhang YL Hendriksen S. Using standardized serum creatinine values in the modification of diet in renal disease study equation for estimating glomerular filtration rate. Ann Intern Med (2006) 145:247–54. doi: 10.7326/0003-4819-145-4-200608150-00004 16908915

[23] Alfonso F Byrne RA Rivero F Kastrati A. Current treatment of in-stent restenosis. J Am Coll Cardiol (2014) 63:2659–73. doi: 10.1016/j.jacc.2014.02.545 24632282

[24] Baigent C Blackwell L Emberson J Holland LE Reith C Bhala N. Efficacy and safety of more intensive lowering of LDL cholesterol: a meta-analysis of data from 170,000 participants in 26 randomised trials. Lancet. (2010) 376:1670–81. doi: 10.1016/s0140-6736(10)61350-5 PMC2988224 21067804

[25] Cannon CP Blazing MA Giugliano RP McCagg A White JA Theroux P. Ezetimibe added to statin therapy after acute coronary syndromes. N Engl J Med (2015) 372:2387–97. doi: 10.1056/NEJMoa1410489 26039521

[26] Sabatine MS Giugliano RP Keech AC Honarpour N Wiviott SD Murphy SA. Evolocumab and clinical outcomes in patients with cardiovascular disease. N Engl J Med (2017) 376:1713–22. doi: 10.1056/NEJMoa1615664 28304224

[27] Ridker PM Everett BM Thuren T MacFadyen JG Chang WH Ballantyne C. Antiinflammatory therapy with canakinumab for atherosclerotic disease. N Engl J Med (2017) 377:1119–31. doi: 10.1056/NEJMoa1707914 28845751

[28] Ray KK Colhoun HM Szarek M Baccara-Dinet M Bhatt DL Bittner VA. Effects of alirocumab on cardiovascular and metabolic outcomes after acute coronary syndrome in patients with or without diabetes: A prespecified analysis of the ODYSSEY OUTCOMES randomised controlled trial. Lancet Diabetes Endocrinol (2019) 7:618–28. doi: 10.1016/s2213-8587(19)30158-5 31272931

[29] Mechanick JI Farkouh ME Newman JD Garvey WT. Cardiometabolic-based chronic disease, adiposity and dysglycemia drivers: JACC state-of-the-Art review. J Am Coll Cardiol (2020) 75:525–38. doi: 10.1016/j.jacc.2019.11.044 PMC7187687 32029136

[30] Gast KB Tjeerdema N Stijnen T Smit JW Dekkers OM. Insulin resistance and risk of incident cardiovascular events in adults without diabetes: meta-analysis. PloS One (2012) 7:e52036. doi: 10.1371/journal.pone.0052036 23300589 PMC3532497

[31] Rewers M Zaccaro D D'Agostino R Haffner S Saad MF Selby JV. Insulin sensitivity, insulinemia, and coronary artery disease: The insulin resistance atherosclerosis study. Diabetes Care (2004) 27:781–7. doi: 10.2337/diacare.27.3.781 14988302

[32] Tenenbaum A Motro M Fisman EZ Adler Y Shemesh J Tanne D. Effect of bezafibrate on incidence of type 2 diabetes mellitus in obese patients. Eur Heart J (2005) 26:2032–8. doi: 10.1093/eurheartj/ehi310 15872029

[33] Simental-Mendía LE Rodríguez-Morán M Guerrero-Romero F. The product of fasting glucose and triglycerides as surrogate for identifying insulin resistance in apparently healthy subjects. Metab Syndr Relat Disord (2008) 6:299–304. doi: 10.1089/met.2008.0034 19067533

[34] Wu S Liu W Ma Q Yu W Guo Y Zhao Y. Association between insulin resistance and coronary plaque vulnerability in patients with acute coronary syndromes: Insights from optical coherence tomography. Angiology. (2019) 70:539–46. doi: 10.1177/0003319718809931 30384773

[35] Bonora E Kiechl S Willeit J Oberhollenzer F Egger G Meigs JB. Insulin resistance as estimated by homeostasis model assessment predicts incident symptomatic cardiovascular disease in caucasian subjects from the general population: The bruneck study. Diabetes Care (2007) 30:318–24. doi: 10.2337/dc06-0919 17259501

[36] Kedhi E Kennedy MW Maehara A Lansky AJ McAndrew TC Marso SP. Impact of TCFA on unanticipated ischemic events in medically treated diabetes mellitus: Insights from the PROSPECT study. JACC Cardiovasc Imaging (2017) 10:451–8. doi: 10.1016/j.jcmg.2015.12.023 27372016

[37] Sánchez-Íñigo L Navarro-González D Fernández-Montero A Pastrana-Delgado J Martínez JA. The TyG index may predict the development of cardiovascular events. Eur J Clin Invest (2016) 46:189–97. doi: 10.1111/eci.12583 26683265

[38] Lambrinoudaki I Kazani MV Armeni E Georgiopoulos G Tampakis K Rizos D. The TyG index as a marker of subclinical atherosclerosis and arterial stiffness in lean and overweight postmenopausal women. Heart Lung Circ (2018) 27:716–24. doi: 10.1016/j.hlc.2017.05.142 28690023

[39] Shi W Xing L Jing L Tian Y Yan H Sun Q. Value of triglyceride-glucose index for the estimation of ischemic stroke risk: Insights from a general population. Nutr Metab Cardiovasc Dis (2020) 30:245–53. doi: 10.1016/j.numecd.2019.09.015 31744716

[40] Thomas B. The global burden of diabetic kidney disease: Time trends and gender gaps. Curr Diabetes Rep (2019) 19:18. doi: 10.1007/s11892-019-1133-6 30826889

[41] Sung KC Park HY Kim MJ Reaven G. Metabolic markers associated with insulin resistance predict type 2 diabetes in koreans with normal blood pressure or prehypertension. Cardiovasc Diabetol (2016) 15:47. doi: 10.1186/s12933-016-0368-7 27001495 PMC4802716

[42] Ho CK Rahib L Liao JC Sriram G Dipple KM. Mathematical modeling of the insulin signal transduction pathway for prediction of insulin sensitivity from expression data. Mol Genet Metab (2015) 114:66–72. doi: 10.1016/j.ymgme.2014.11.003 25468647 PMC4319670

[43] Laxy M Hunger M Stark R Meisinger C Kirchberger I Heier M. The burden of diabetes mellitus in patients with coronary heart disease: A methodological approach to assess quality-adjusted life-years based on individual-level longitudinal survey data. Value Health (2015) 18:969–76. doi: 10.1016/j.jval.2015.07.003 26686780

[44] Quiroga B Muñoz Ramos P Sánchez Horrillo A Ortiz A Valdivieso JM Carrero JJ. Triglycerides-glucose index and the risk of cardiovascular events in persons with non-diabetic chronic kidney disease. Clin Kidney J (2022) 15:1705–12. doi: 10.1093/ckj/sfac073 PMC9394724 36003671

[45] Şaylık F Çınar T Selçuk M Tanboğa İH. The predictive value of triglyceride-glucose index for in-hospital and one-year mortality in elderly non-diabetic patients with ST-segment elevation myocardial infarction J Geriatr Cardiol (2022) 19:610–7 doi: 10.11909/j.issn.1671-5411.2022.08.006 PMC9630000 36339471

