# Supplementary material for: High triglyceride–glucose index is associated with poor cardiovascular outcomes in Chinese acute coronary syndrome patients without diabetes mellitus who underwent emergency percutaneous coronary intervention with drug-eluting stents
Source: Front Endocrinol (Lausanne). 2023 Feb 15;14:1101952. doi: 10.3389/fendo.2023.1101952 (PMC9975349; doi:10.3389/fendo.2023.1101952)
Supplement: Supplementary file 1 [file Table_1.docx]

**Supplemental Table 1 Baseline clinical characteristics of patients with and without MACCE groups**

| Characteristics | Total population | Without MACCE group | With MACCE group | *P* value |
| --- | --- | --- | --- | --- |
|  | (n=1650) | (n=1213) | (n=437) |  |
| Age, years | 60.50 ± 10.19 | 60.30 ± 10.08 | 61.05 ± 10.50 | 0.191 |
| Gender, male, n (%) | 1279（77.5%） | 926（76.3%） | 353（80.8%） | 0.045 |
| BMI, kg/m2 | 23.26 ± 2.46 | 23.26 ± 2.46 | 23.27 ± 2.45 | 0.955 |
| SBP, mmHg | 126.93 ± 20.74 | 126.67 ± 20.99 | 127.64 ± 20.03 | 0.407 |
| DBP, mmHg | 77.67 ± 11.97 | 77.51 ± 12.03 | 78.11 ± 11.81 | 0.366 |
| Heart rate, bpm | 73.91 ± 12.05 | 73.68 ± 12.22 | 74.55 ± 11.54 | 0.197 |
| Smoking history, n (%) | 928（56.2%） | 664（54.7%） | 264（60.4%） | 0.053 |
| Drinking history, n (%) | 459（27.8%） | 357（29.4%） | 102（23.4%） | 0.016 |
| Family history of CAD, n (%) | 145（8.8%） | 103（8.5%） | 42（9.6%） | 0.467 |
| Initial diagnosis, n (%) |  |  |  | 0.009 |
| UA | 976（59.2%） | 746（61.5%） | 230（52.6%） |  |
| NSTEMI | 118（7.2%） | 91（7.5%) | 27（6.2%） |  |
| STEMI | 556（33.7%） | 376（31.0%） | 180（41.2%） |  |
| Medical history, n (%) |  |  |  |  |
| Hypertension | 886（53.7%） | 641（52.8%） | 245（56.2%） | 0.223 |
| Dyslipidemia | 180（10.9%） | 121（10.0%） | 59（13.5%） | 0.059 |
| Renal dysfunction | 14（0.8%） | 8（0.7%） | 6（1.4%） | 0.161 |
| Previous MI | 148（9.0%） | 103（8.5%） | 45（10.3%） | 0.250 |
| Previous PCI | 158（9.6%） | 108（8.9%） | 50（11.5%） | 0.118 |
| Previous stroke | 321（19.5%） | 241（19.9%） | 80（18.3%） | 0.496 |
| Previous PAD | 260（15.8%） | 190（15.7%） | 70（16.1%） | 0.842 |
| Laboratory results |  |  |  |  |
| WBC ( ×109/L) | 7.30 ± 2.57 | 7.15 ± 2.55 | 7.36 ± 2.57 | 0.156 |
| PLT ( ×109/L) | 158.49 ± 55.42 | 158.89 ± 53.82 | 157.36 ± 59.69 | 0.621 |
| Hb (g/L) | 138.15 ± 18.40 | 138.87 ± 18.41 | 136.14 ± 18.25 | 0.008 |
| BUN (mmol/L) | 5.29 ± 1.83 | 5.27 ± 1.78 | 5.37 ± 1.97 | 0.324 |
| Cr (umol/L) | 67.93 ± 17.72 | 67.70 ± 16.99 | 68.58 ± 19.60 | 0.407 |
| eGFR (mL/min/1.73m2) | 97.48 ± 27.25 | 97.42 ± 26.53 | 97.66 ± 29.21 | 0.875 |
| FBG (mmol/L) | 5.92 ± 1.85 | 5.83 ± 1.80 | 6.15 ± 1.94 | 0.003 |
| HbA1C (%) | 5.61 ± 0.64 | 5.60 ± 0.60 | 5.65 ± 0.74 | 0.178 |
| HDL-C (mmol/L) | 0.98 ± 0.23 | 0.99 ± 0.23 | 0.96 ± 0.22 | 0.059 |
| TC (mmol/L) | 3.71 ± 1.17 | 3.72 ± 1.15 | 3.69 ± 1.21 | 0.611 |
| TG (mmol/L) | 1.52 ± 0.90 | 1.49 ± 0.88 | 1.60 ± 0.95 | 0.041 |
| LDL-C (mmol/L) | 1.82 ± 0.85 | 1.80 ± 0.83 | 1.85 ± 0.89 | 0.278 |
| NT-proBNP (pg/mL) | 690.55 ± 1233.19 | 680.67 ± 1166.87 | 718.04 ± 1402.52 | 0.587 |
| TyG index | 7.13 ± 0.54 | 7.10 ± 0.52 | 7.20 ± 0.60 | 0.001 |
| LVEF (%) | 60.07 ± 11.28 | 60.02 ± 11.08 | 60.23 ± 11.81 | 0.735 |
| Angiographic data |  |  |  |  |
| LM disease, n (%) | 174（10.5%） | 116（9.6%） | 58（13.3%） | 0.029 |
| CTO, n (%) | 475（28.8%） | 345（28.4%） | 130（29.8%） | 0.580 |
| Number-vessel disease. n (%) |  |  |  | 0.061 |
| Single-vessel disease | 445（27.0%） | 338（27.8%） | 107（24.5%） |  |
| Two-vessel disease | 497（30.1%） | 375（30.9%） | 122（28.0%） |  |
| Three-vessel disease | 708（42.9%） | 500（41.2%） | 208（47.7%） |  |
| Diffuse lesion, n (%) | 985（59.7%） | 725（59.7%） | 260（59.6%） | 0.975 |
| In-stent restenosis, n (%) | 48（2.9%） | 36（3.0%） | 12（2.8%） | 0.820 |
| Calcification lesion, n (%) | 38（2.3%） | 30（2.5%） | 8（1.8%） | 0.447 |
| Number of stents | 1.71 ± 1.15 | 1.68 ± 1.12 | 1.82 ± 1.23 | 0.029 |
| Medication at discharge, n (%) |  |  |  |  |
| ACEI/ARB | 1345（81.5%） | 984（81.1%） | 361（82.8%) | 0.421 |
| β-blocker | 1338（81.1%） | 998（82.3%） | 340（77.8%） | 0.039 |
| Statins | 1643（99.6%） | 1207（99.4%） | 436（100.0%） | 0.246 |
| P2Y_12_ inhibitor |  |  |  | 0.343 |
| Clopidogrel | 1491（90.4%） | 1092（90.0%） | 399（91.3%） |  |
| Ticagrelor | 159（9.6%） | 121（10.0%） | 38（8.7%） |  |
| Aspirin | 1650（100.0%） | 825（100%） | 825（100%） | - |
| DAPT | 1650（100.0%） | 825（100%） | 825（100%） | - |

*MACCE: major adverse cardiovascular and cerebrovascular events; BMI: body mass index; SBP: systolic blood pressure; DBP: diastolic blood pressure; CAD: coronary artery disease; UA: unstable angina; NSTEMI: non ST-segment elevation myocardial infarction; STEMI: ST-segment elevation myocardial infarction; MI: myocardial infarction; PCI: percutaneous coronary intervention; PAD: peripheral artery disease; WBC: white blood cell; PLT: platelet; Hb: hemoglobin; BUN: blood urea nitrogen; Cr: creatinine; eGFR, estimated glomerular filtration rate; FBG: fasting blood glucose; HbA1C: glycosylated hemoglobin A1c; HDL-C: high-density lipoprotein cholesterol; TC: total cholesterol; TG: triglyceride; LDL-C: low-density lipoprotein cholesterol; TyG: triglyceride-glucose; LVEF: left ventricular ejection fraction; LM: left main; CTO: chronic total occlusion; ACEI: angiotensin converting enzyme inhibitor; ARB: angiotensin receptor blocker; DAPT: dual antiplatelet therapy.*
